# Occurrence of Glioblastoma Five Years After Resection of Atypical Meningioma at the Same Anatomic Site: A Case Report

**DOI:** 10.7759/cureus.66871

**Published:** 2024-08-14

**Authors:** Gerardo Romero-Luna, Andres Ludwig Mendez-Granda, Raul Adame-Paredes, Gabriel Galvan-Salazar, Pedro Pablo De Juambelz-Cisneros, Basilio Fernandez-Alvarado, Fernando Pazos-Gómez

**Affiliations:** 1 Neurosurgery, Instituto Nacional de Neurología y Neurocirugía, Mexico City, MEX; 2 General Surgery, Hospital Español de México, Mexico City, MEX; 3 Oncology, Hospital Español de México, Mexico City, MEX; 4 Neurosurgery, Hospital Español de México, Mexico City, MEX

**Keywords:** radio-neurosurgery, craniectomy, asynchronous brain tumor, glioblastoma, atypical meningioma

## Abstract

Atypical meningioma is a type of intermediate-grade meningioma (grade 2) according to the WHO classification. The occurrence of glioblastomas at the same site of resection of a meningioma is extremely rare and the causes of this type of mutations should be investigated. We present a case of a 54-year-old patient who five years after resection of a left parietooccipital atypical meningioma presented with a glioblastoma at the same site.

## Introduction

Meningiomas are the most common primary tumors of the central nervous system. According to the new WHO classification of meningiomas, they are divided into three grades, with atypical, chordoid, and clear cell meningiomas being of intermediate grade (grade 2) [1]. Glioblastoma is the most common primary malignant tumor of the central nervous system, and complete resection of this tumor has so far been practically impossible [2]. Over the years, an increase in the survival of these patients has been observed, being 18 months as the standard survival after the treatment with surgery and radiosurgery [3]. Synchronous and asynchronous occurrence of glioblastomas at previous meningioma resection sites is extremely rare, and these patients should be subject to investigation. We report the case of a patient with a history of resection of atypical meningioma with subsequent occurrence of glioblastoma at the same site.

## Case presentation

**Figure 1:**
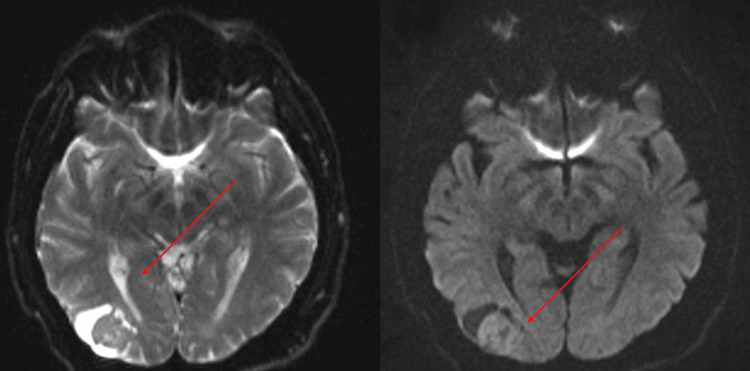
Right occipital lesion, occipital, heterogeneous, cystic in appearance with a solid component inside.

A 54-year-old male patient started presenting mild symptoms in 2019 with a feeling of dizziness, palpitations, weakness in the lower limbs, and blurred vision, later presenting with a generalized tonic-clonic epileptic seizure and fracture of both humeri, for which a brain tomography and contrasted magnetic resonance imaging was performed with the finding of a right occipital lesion of extra-axial appearance (Figure 1).

**Figure 2:**
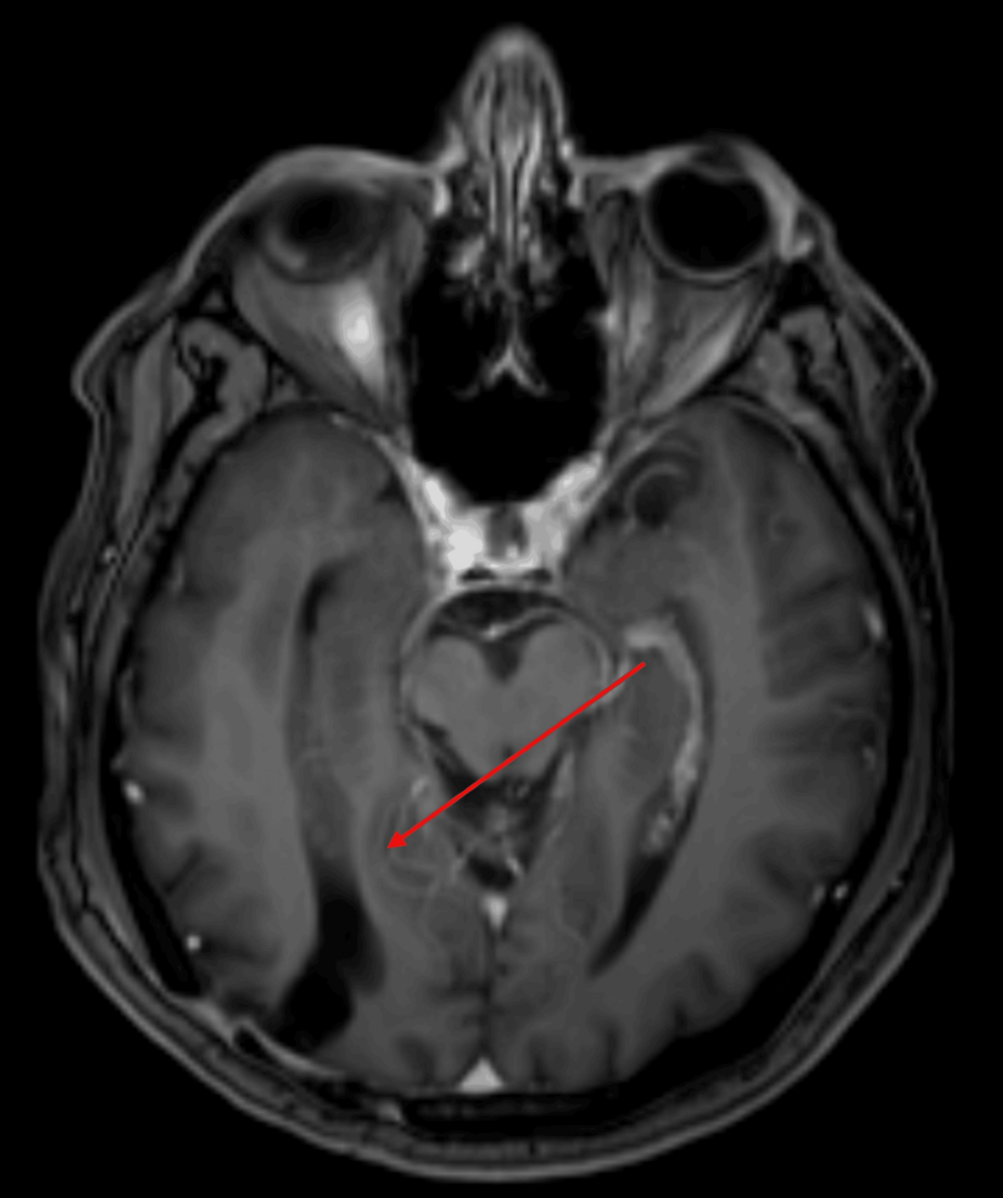
Axial MRI with areas of the right occipitoparietal gliosis without evidence of lesion recurrence.

In view of these findings, a right parietooccipital craniectomy + total resection of the lesion was performed showing an extra-axial lesion with histopathological findings of right parietooccipital atypical meningioma grade 2, with no complications after surgery, although he did not receive any additional treatment, such as chemotherapy or radiosurgery. After surgery, a follow-up with a cranial MRI was performed in 2020, with no apparent recurrence of the lesion (Figure 2).

**Figure 3:**
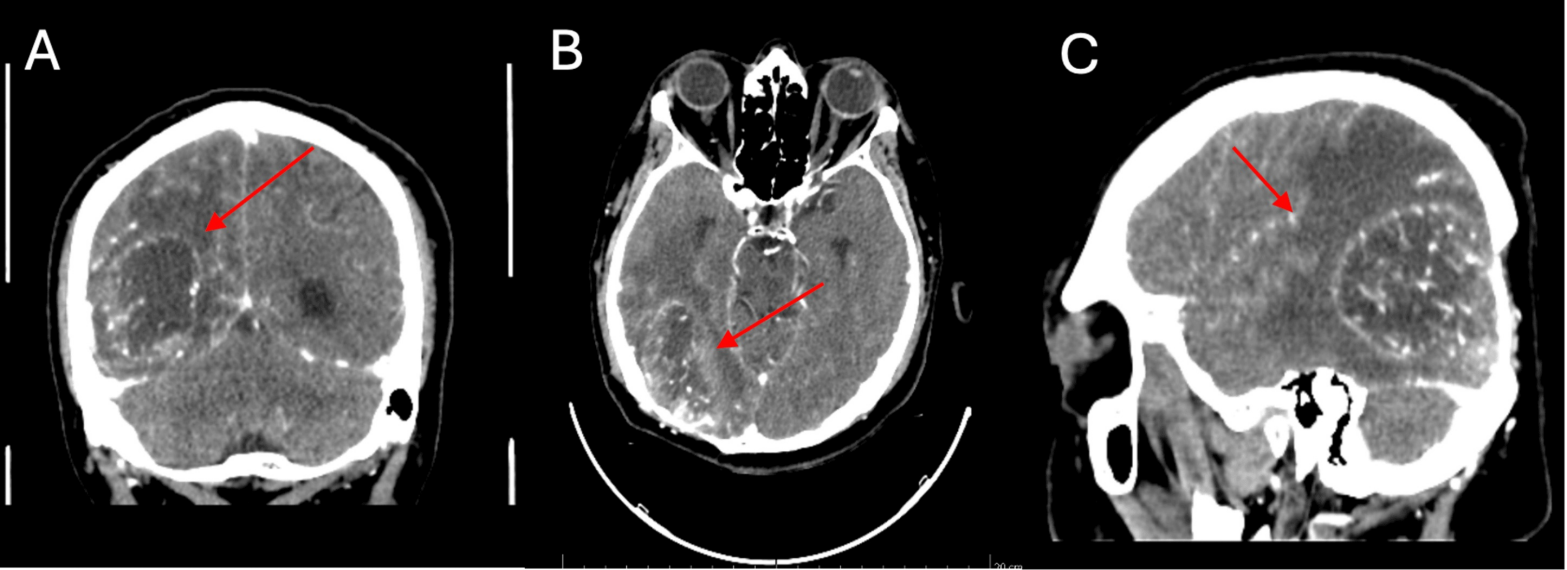
Brain CT scan with the presence of a heterogeneous space-occupying lesion, right parietooccipital, with extensive perilesional edema, multiple hypointense vascular structures inside, with moderate enhancement, irregular contours. A. Coronal section. B. Axial view. C. Sagittal view

In June 2024, he attended a follow-up appointment with an oppressive headache, bilateral frontal headache, and diplopia, so a cranial tomography with contrast was requested with an image suggestive of glioblastoma (Figure 3). Thus, it was decided to admit him again for resection of the new lesion.

The surgical procedure was performed consisting of right parietooccipital craniectomy guided by neuronavigation, partial excision, and fluorescein-guided biopsy, reporting a grade 4 glioblastoma IDH-wildtype central nervous system (CNS) WHO 4 with a proliferation index of 10%, so it was decided to continue treatment with radiosurgery and neurological surveillance.

**Figure 4:**
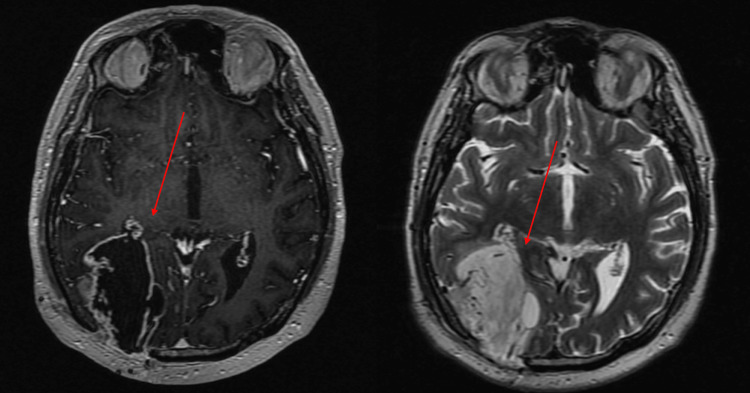
A. Cranial MRI in contrast-enhanced T1-weighted sequence with the presence of the right parietooccipital lesion with a halo uptake of the contrast medium. B. Cranial MRI in T2 sequence with evidence of the lesion with a cystic portion, well delimited, without the presence of peritumoral edema.

The patient attended a follow-up consultation with a contrasted cranial MRI for the evaluation of the lesion and initiation of radiosurgical treatment (Figure 4).

## Discussion

There are very few reports in the literature on the asynchronous appearance of glioblastomas in meningioma resection sites, while still not common, the synchronous appearance of both tumors is more frequently documented [4]. According to the histopathologic study, meningiomas should have three or more of the histologic features shown in Table 1, as well as 4-19 mitoses per field and direct invasion into the brain parenchyma (invade at least the pia mater).

**Table 1:** Characteristics of atypical meningioma.

Characteristics of atypical meningioma
4-19 mitoses per HPF
3 or more of the following histological features: 1. Necrosis, 2. Layered growth, 3. Small cell changes, 4. Increased cellularity, 5. Prominent nucleolus.
Direct parenchymal invasion

The resection of these tumors should be performed with a Simpson grade 1 or 2 and subsequent radiotherapy treatment to avoid recurrence, as in our case in which a Simpson 1 resection was performed [5]. In cases of glioblastoma, there is a protein called isocitrate dehydrogenase (IDH), which, according to the latest WHO CNS 5 classification, there is no IDH-mutant glioblastoma, only IDH-wildtype glioblastoma or IDH-mutant astrocytoma. In the case we present, the patient had an IDH wildtype glioblastoma, making resection more complicated and with less response to radiotherapy, even though he was a candidate for it [6].

In the literature, there are some reports in which there may be a collision tumor, in which two different types of tumors are found simultaneously. In our case, the first tumor resection found only atypical meningioma without the presence of cellularity or markers suggestive of glioblastoma, so we ruled out this diagnosis, especially since the treatments for these conditions are completely different [7].

Similarly, the form of presentation is usually different, since meningiomas refer to extra-axial tumors that cause compressive symptoms as opposed to glioblastomas, which are intra-axial tumors that usually cause focalization. Similarly, there are certain meningiomas that, by imaging or intraoperative studies, can mimic a glioblastoma, with characteristics such as high vascularity, central necrosis, and invasion of margins without apparent dural invasion, among others [8]. In the case presented, the patient showed high vascularity of the lesion with adherence to meninges, without presenting other characteristics of glioblastoma, but the diagnosis was confirmed with the histopathological study.

## Conclusions

The development of a grade 4 glioma at the same site following resection of a meningioma is extremely rare, and a more extensive analysis of the issue should be done to know the cause of these mutations, whether caused by radiosurgery or other internal and external factors that could be present in these patients.
